# Advances of Carbon Materials for Dual-Carbon Lithium-Ion Capacitors: A Review

**DOI:** 10.3390/nano12223954

**Published:** 2022-11-10

**Authors:** Ying Duan, Changle Li, Zhantong Ye, Hongpeng Li, Yanliang Yang, Dong Sui, Yanhong Lu

**Affiliations:** 1 Henan Key Laboratory of Function-Oriented Porous Materials, College of Chemistry and Chemical Engineering, Luoyang Normal University, Luoyang 471934, China; 2 College of Food and Drug, Luoyang Normal University, Luoyang 471934, China; 3 School of Chemistry & Material Science, Langfang Normal University, Langfang 065000, China; 4 College of Mechanical Engineering, Yangzhou University, Yangzhou 225127, China

**Keywords:** lithium-ion capacitor, nanocarbon, carbon nanomaterial, dual-carbon electrode, high energy/power density

## Abstract

Lithium-ion capacitors (LICs) have drawn increasing attention, due to their appealing potential for bridging the performance gap between lithium-ion batteries and supercapacitors. Especially, dual-carbon lithium-ion capacitors (DC-LICs) are even more attractive because of the low cost, high conductivity, and tunable nanostructure/surface chemistry/composition, as well as excellent chemical/electrochemical stability of carbon materials. Based on the well-matched capacity and rate between the cathode and anode, DC-LICs show superior electrochemical performances over traditional LICs and are considered to be one of the most promising alternatives to the current energy storage devices. In particular, the mismatch between the cathode and anode could be further suppressed by applying carbon nanomaterials. Although great progresses of DC-LICs have been achieved, a comprehensive review about the advances of electrode materials is still absent. Herein, in this review, the progresses of traditional and nanosized carbons as cathode/anode materials for DC-LICs are systematically summarized, with an emphasis on their synthesis, structure, morphology, and electrochemical performances. Furthermore, an outlook is tentatively presented, aiming to develop advanced DC-LICs for commercial applications.

## 1. Introduction

Rechargeable energy storage systems are indispensable energy supply components for consumer electronics, electric vehicles, and smart grids [1,2,3,4,5]. More importantly, the reduction of greenhouse gas and the effective utilization of sustainable and clean, but intermittent, solar and wind energy also depend on the development of high-performance energy conversion and storage systems [6,7,8,9]. Generally, lithium-ion batteries (LIBs) and supercapacitors (SCs) are the two commonly used energy storage devices and exhibit different electrochemical performances, due to the different energy storage mechanisms. LIBs store/release energy through lithium ions rocking back and forth reversibly between a cathode and anode via a Faradaic reaction, thereby delivering high energy density and high working voltage, as well as low self-discharge [10,11,12]. By contrast, SCs have the advantages of high power density and long cycling life, based on a fast, non-Faradaic adsorption/desorption mechanism [13]. Nevertheless, both LIBs and SCs have their own shortcomings. For example, LIBs suffer from inferior power performance and limited lifespan, which are ascribed to the severely sluggish Li^+^ diffusion in the bulk electrode and structural degradation during repeated charge/discharge processes, respectively [14]. As for the SCs, the physically electrostatic ion adsorption at the electrode/electrolyte interface coupled with small working voltage results in relatively lower energy density, thus hindering their further applications [15]. Therefore, there is an urgent need to develop high-performance energy storage devices that can combine the high energy/working voltage of LIBs with the high power/long lifespan of SCs.

**Figure 1:**
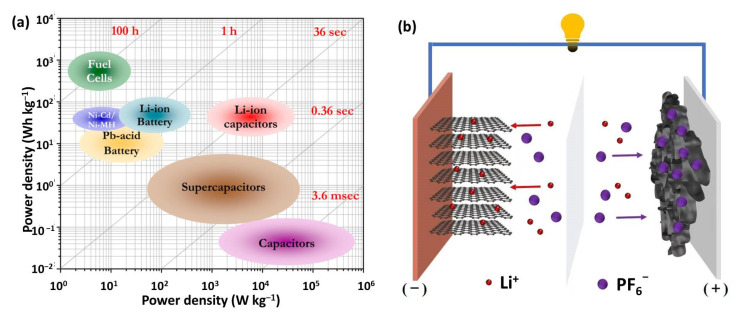
(**a**) Ragone plot of various energy storage devices. Reproduced with permission from Ref. [20]. Copyright 2014, American Chemical Society. (**b**) The configuration and energy storage mechanism of LICs.

To solve the above-mentioned disadvantages of LIBs and SCs, lithium-ion capacitors (LICs, also named lithium-ion hybrid supercapacitors) offer a promising solution by integrating the advantages of LIBs and SCs into one system. LICs possess not only higher power density than LIBs and other conventional batteries, but also higher energy density than SCs (Figure 1a). As for the typical device configuration, LICs are composed of a capacitor-type electrode from SCs as the cathode to ensure high power output and a battery-type electrode from LIBs as the anode to provide high energy density (Figure 1b) [16]. Lithium salts dissolved in organic solvents are commonly adopted as the electrolytes, delivering a wide working voltage [17]. Compared with LIBs and SCs, LICs follow a hybrid energy storage mechanism. During the charge/discharge process, lithium ions intercalate/de-intercalate into/from the anode through a redox reaction, while anions absorb/desorb onto/from the cathode through formation of an electric double layer at the electrode–electrolyte interface [18]. It is noted that LICs could deliver a much higher output voltage than that of SCs because the anode and cathode work in different potential regions [19]. Therefore, LICs with a hybrid cell configuration can well-bridge the performance gap between LIBs and SCs. Benefiting from the high energy and high power density, LICs have shown great potential in start power, electric vehicles, and frequency modulation in grid and large-scale energy storage systems. So far, LICs have received intensive research interest from both the academic and industrial communities.

Since the electrochemical performances of LICs are significantly influenced by the capacity, rate capability, operation potential, and cycling stability of both the anode and cathode, developing advanced electrode materials is of great importance [17,21,22]. Notably, nanostructured materials have a positive effect on the electrochemical properties of an electrode. For example, a nanosized anode has a better rate, has a longer cycling life, and is a better match with the cathode in kinetics. On the other hand, a cathode with rationally distributed nanoscaled pores has a higher capacity. So far, various cathode and anode materials with stable structure, tailorable microstructure, and rich surface chemistry and matched properties have been investigated in LICs. The capacitor-type cathode is expected to ensure high power and long-term durability, so that porous carbon materials with high electrical conductivity, large specific surface area (SSA), and rich nanosized pores are commonly adopted [22]. To realize high energy and power density, nanosized carbons, such as graphene, carbon nanotubes (CNTs), and their composites with inherently high conductivity and large SSA, have commonly been investigated recently because of the high capacity and excellent rate [23,24]. As compared with the cathode, the battery-type anode materials are the key components to supply high energy. According to the lithium storage mechanisms, anode materials can be roughly divided into three categories: intercalation-type materials (carbonaceous materials, Ti/Nb-based compounds, etc.), conversion-type materials (metal oxide/sulfide/nitride, etc.), and alloying-type materials (Si/Sn-based materials, etc.) [25,26,27,28,29]. LICs using non-carbon materials as anodes usually deliver one superior property at the cost of other performances. For instance, LICs with Ti/Nb-based anodes show high power density and excellent cycling stability, due to their fast Li^+^ intercalation/de-intercalation rate and stable structure [30,31,32]. However, they have a lower specific capacity and higher Li^+^ insertion/de-insertion potential than other anodes, leading to unsatisfactory energy density. Although LICs based on conversion/alloying-type anodes could realize high energy density, their intrinsically low conductivity and large volume change during charge/discharge process always result in poor power density and limited cycling life [33,34,35,36]. To overcome the above-mentioned drawbacks of non-carbon anodes, developing nanostructured materials, introducing defects, and compositing with nanosized carbons are typically the adopted strategies to enhance the conductivity and/or suppress the volume change [37,38,39]. Under these circumstances, carbon anodes have drawn inceasing attention because carbon possesses many superior properties over others. The excellent electrical conductivity, relatively high capacity, and stable structure of carbon materials enable LICs with not only high energy/power densities, but also outstanding cycling stability. In addition, the abundant resources, mature material production, and device fabrication technologies of carbon-based materials could promote their commercial application in LICs at a low cost.

**Figure 2:**
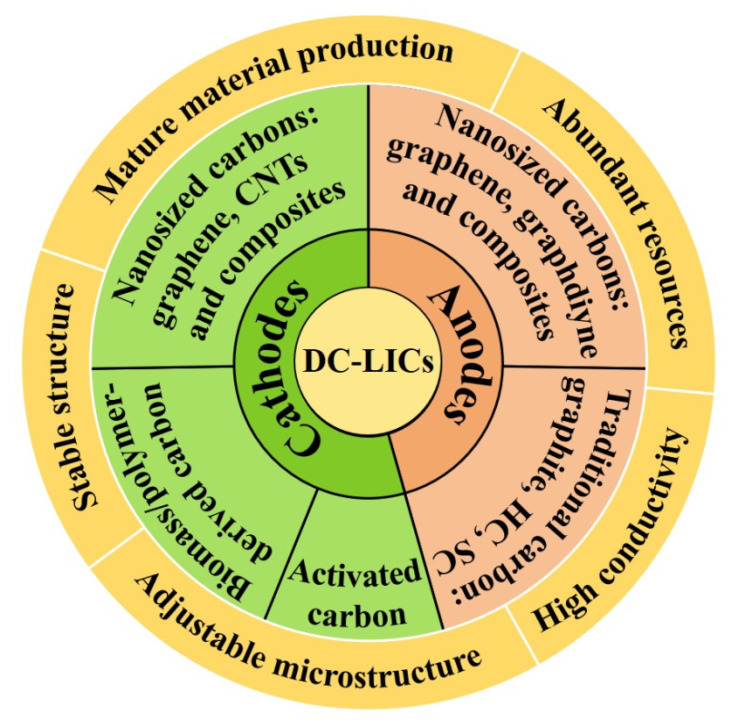
Typical carbon cathode and anode materials for DC-LICs.

Compared with non-carbon anode materials, the carbon anode could well-match with the porous carbon cathode, in terms of capacity, rate, and cycle life. Therefore, tremendous efforts have been devoted to developing dual-carbon lithium-ion capacitors (DC-LICs) by adopting carbon materials as both the cathode and anode (Figure 2). Similar to the typical LICs, cathode materials for DC-LICs are activated carbon (AC), biomass/polymer-derived porous carbons, and nanosized carbons (graphene, CNTs, and composites) [23,40,41,42]. The anode materials of DC-LICs include traditional carbon materials (graphite, hard carbon, and soft carbon), and nanosized carbons (graphene, CNTs, graphdiyne, and their composites) [22,37,43]. Benefitting from the low cost, abundance in raw materials, tunable surface chemistry and composition, adjustable structure and morphology, and excellent physical/chemical/electrochemical stability of carbon materials, DC-LICs have demonstrated superior electrochemical performances. It should be noted that nanocarbon-based DC-LICs show superior electrochemical properties over the traditional carbons because of the enhanced capacity and rate capability [13,19,44]. During the past two decades, great progresses of electrode materials, electrolytes, cell configuration, and pre-lithiation technology for LICs have been achieved and extensively reviewed [22,24,45,46,47]. However, a systematical summary of the advances in electrode materials for DC-LICs is highly needed [48,49]. Therefore, in this review, the latest progresses of electrode materials ranging from traditional carbons to novel nanosized carbons for DC-LICs are comprehensively overviewed. The material synthesis, structure, morphology, electrochemical performance, and merits and demerits are particularly discussed, aiming to develop high-performance carbon-based electrode materials for industrial-scale applications. Furthermore, the outlooks for DC-LICs are tentatively presented. This review will provide some constructive guidelines for fabricating advanced DC-LICs for both the scientific community and industry.

## 2. Progresses of Carbon Materials as Cathode for DC-LICs

Based on the energy storage mechanism of fast ion adsorption/desorption at the electrode/electrolyte interface, the capacitor-type cathodes are expected to provide high power density and ensure long-term durability in LICs. Bearing this in mind, porous carbon materials with high SSA, rich interconnected pores, excellent electrical conductivity, and stable structure are investigated as cathodes. Thus, traditional porous carbons (AC, biomass/polymer-derived porous carbon) and novel nanosized carbons (graphene, CNTs and their composites) are commonly adopted. The typical examples are summarized in Table 1.

### 2.1. Traditional Porous Carbon Cathode

#### 2.1.1. Activated Carbon

As a conventional porous carbon material used in SCs, AC has the advantages of acceptable SSA, porous structure, low cost, and mature fabrication technology. Typically, ACs are prepared from carbon-rich materials through activation with KOH, H_3_PO_4_, ZnCl_2_, H_2_O, and/or CO_2_ at high temperature [73]. In the early studies of LICs, AC was usually used as the cathode. For example, the first protype of LIC was fabricated with AC as the cathode and nanostructured Li_4_Ti_5_O_12_ as the anode [74]. The obtained asymmetric hybrid system shows high energy density, an extended cycle life, and fast charge/discharge capability. Later, several groups developed DC-LICs by pairing AC with graphite [75], hard carbon [40,76], or graphene [77]. However, conventional ACs still suffer from low capacity and poor rate capability, due to small SSA and low conductivity, resulting in inferior energy and power densities. Hence, it is highly urgent to develop advanced ACs with high conductivity and desired microstructure.

To further improve the capacity and rate of AC, compositing with nanosized carbons is a feasible method. For example, Ma et al. designed graphene/activated carbon (G/AC) composites through a fast, self-propagating, high-temperature synthesis (SHS) process, which combined the advantages of the two components [78]. The conductivity of the prepared G/AC was largely enhanced from 389 to 2941 S m^−1^, and a remarkable rate performance with 84% capacity retention at 10 A g^−1^ was achieved, compared with 65% for pure AC. Based on this cathode, the assembled LICs with graphene/soft carbon anode demonstrated a greatly improved energy density of 152 Wh kg^−1^ and power density of 18.9 kW kg^−1^.

#### 2.1.2. Biomass-Derived Porous Carbon

Recently, biomass-derived porous carbons (BDPCs) are emerging as cost-effective electrode materials for DC-LICs, due to their abundance, renewability, and sustainability [53,79]. Biomass is an economical and environmentally friendly raw material for preparing porous carbon and has the following merits: (1) the diversity of biomass with different nanostructure and morphology provides many choices for preparing porous carbon with desired electrochemical performances; (2) the inherent ordered porous structure of biomass facilitates the activation process to form porous carbon with high SSA and large pore volume; (3) the heteroatom doped porous carbon could be facilely realized via a self-doping strategy [80,81,82]. Particularly, heteroatom doping has multiple advantages. The study by Lee et al. revealed that heteroatom doping is beneficial to the betterment of electrical conductivity and pore generation, which eventually enhances the electrochemical properties of carbonaceous materials [83].

**Figure 3:**
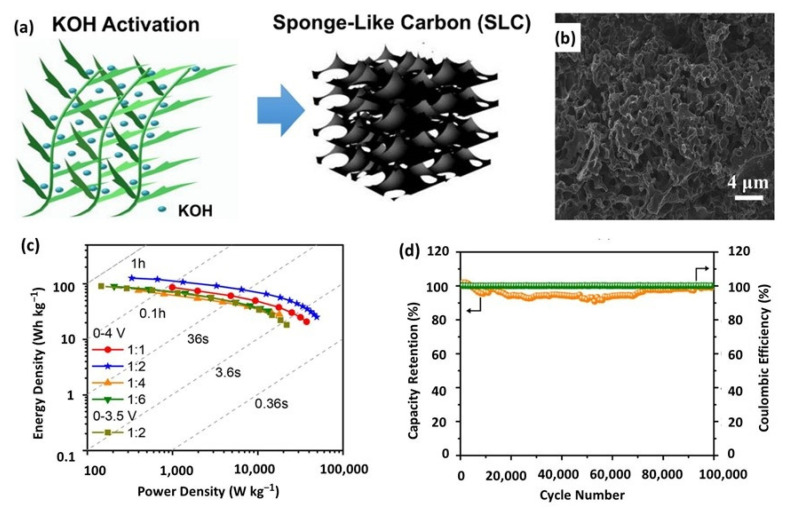
(**a**) Schematic illustration of the preparation process of SLC; (**b**) SEM image of SLC; (**c**) Ragone plots and (**d**) cycling stability of DC-LICs based on SLC electrodes. Reproduced with permission from Ref. [91]. Copyright 2019, American Chemical Society.

Similar to conventional ACs, BDPCs are commonly prepared via a typical carbonization and activation processes [51,84]. Additionally, BDPCs have porous morphology, stable structure, heteroatom dopants, and high conductivity, endowing SCs or LICs based on them with high energy/power density and good cycling stability [82,84,85]. Several works have explored agricultural waste-derived porous carbons in LICs by pairing with non-carbon anodes, which demonstrate that BDPCs could serve as excellent cathodes [86,87,88,89,90]. Then, the applications of BDPCs in DC-LICs were investigated. Wang et al. prepared a sponge-like carbon (SLC) from gulfweed through KOH activation and used as electrode material for DC-LICs (Figure 3a) [91]. As shown in Figure 3b, the obtained sample exhibited a sponge-like structure with rich porosity. Benefiting from the astonishing structure, rational pore size distribution (PSD), and heteroatom doping, the optimized device with SLC as both cathode and anode delivered a high energy density of 127 Wh kg^−1^ and a peak power of 33.57 kW kg^−1^ (Figure 3c). More importantly, an ultra-stable cycling performance with 99% capacity retention was achieved after 100,000 cycles (Figure 3d). Ma et al. reported a cathode of N-doped hierarchical carbon nanolayer (NHCNs) through a facile, one-step template carbonization/activation from naturally abundant and renewable chitosan biomass [52]. NHCNs has a high capacity of 125 mAh g^−1^, which could be ascribed to its hierarchically porous structure with large SSA (2350 m^2^ g^−1^) and high nitrogen doping level. Moreover, DC-LICs based on NHCNs show a maximum power density of 52 kW kg^−1^ and an ultra-long cycling life of 40,000 cycles. Anyway, graphene is commonly introduced to further enhance the conductivity of BDPCs by forming composites, aiming to achieve higher specific capacity and better rate capability [86,92,93]. It should be noted that biomass-derived carbons have the disadvantages of uncontrollable impurity and element composition, due to the diversity of raw materials. Tedious post treatment is usually needed to obtain products with high purity and good batch stability.

#### 2.1.3. Polymer-Derived Porous Carbon

Porous carbon materials could also be prepared through pyrolysis and the activation of polymers. Compared with the uncontrollable impurity and element composition of biomass-derived carbons, polymers could be designed and adjusted from the precursors, so that the structure and composition could be well-controlled. Thus, polymer-derived porous carbons receive increasing interests in energy storage devices [41,94]. For instance, Hung et al. designed a hierarchical porous activated carbon (H-HPAC) material through pyrolysis of polyvinylpyrrolidone (PVP)-derived hydrogel, using K_2_CO_3_ as both the initiator for hydrogel formation and the activator [95]. In the preparing process, the numerous water molecules captured within the PVP function as a green template during the formation of hydrogel, making this method a facile and eco-friendly strategy to design highly porous carbon. As a cathode, H-HPAC demonstrates a capacitance of 128.7 F g^−1^, due to its high SSA (2012 m^2^ g^−1^) and large pore volume (1.16 cm^3^ g^−1^). Wang et al. proposed a nitrogen-doped activated porous carbon by carbonization and activation of polypyrrole [96]. The product has a hierarchically porous structure with plenty of mesopores created by surfactant and micropores generated by activation. The all-carbon LIC delivers a high energy density of 167 Wh kg^−1^, which still remains 88.9 Wh kg^−1^ at an ultrahigh power density of 13.2 kW kg^−1^. Similarly, Ajuria et al. reported an activated carbon from furfuryl alcohol-based polymer by a process of polymerization, carbonization, and activation [97]. The obtained cathode material showed a sponge-like surface with high SSA and broad pore distribution. Combined with a hard carbon anode made from the same furfuryl alcohol-derived polymer, the obtained DC-LICs offer a medium energy density of 110 Wh kg^–1^ at 7 kW kg^–1^ and keep 50 Wh kg^–1^, even at an ultrahigh power of 50 kW kg^–1^ (discharge in less than 10 s). Although polymer-derived porous carbons have demonstrated advantages over biomass, in terms of purity and controllable composition, the high cost and tedious synthesis process should not be ignored.

As a class of organic porous polymers, conjugated microporous polymers (CMPs), covalent organic frameworks (COFs), and their derivatives have been applied in various energy storage devices [98,99,100,101,102,103]. CMPs and COFs have the merits of well-defined crystal structure, plenty of nanopores, large SSA, and rich heteroatoms, making them the ideal platform to prepare high-performance carbon materials [104,105]. Therefore, CMPs and COFs could serve as excellent precursors to prepare self-doping porous carbons via a simple pyrolysis process. CMPs/COFs-derived porous carbons have been successfully used as electrode materials in LIBs and SCs [106,107,108,109]. However, there are very few reports covering the application of CMPs/COFs-derived porous carbons in LICs or DC-LICs. Hence, more efforts could focus on this field in future research.

### 2.2. Nanosized Carbon-Based Cathode

Traditional AC and biomass/polymer-derived porous carbons usually suffer from low capacity, due to too many inaccessible micropores, resulting in inferior energy density. Moreover, their amorphous structure with numerous pores and defects leads to low electrical conductivity and, thus, poor power output. Therefore, nanosized carbons, such as graphene, CNTs, and their composites, are emerging as excellent cathodes for DC-LICs because of their inherently high conductivity and large theoretical SSA.

#### 2.2.1. Graphene-Based Cathode

Graphene, as a novel two-dimensional (2D) nanosized carbon material, has drawn intensive attention, ever since its discovery and has found numerous applications in energy conversion and storage [110,111,112]. Graphene possesses the following advantages. First of all, the layered structure with long-range conjugation endows graphene with large SSA, high electrical conductivity, and outstanding mechanical strength [113]. Anyway, the rich surface chemistry coupled with the 2D structure also makes graphene an excellent substrate for forming composites with other materials [13,114,115]. As a result, graphene-based materials have been adopted as an excellent cathode in LICs [23,24].

**Figure 4:**
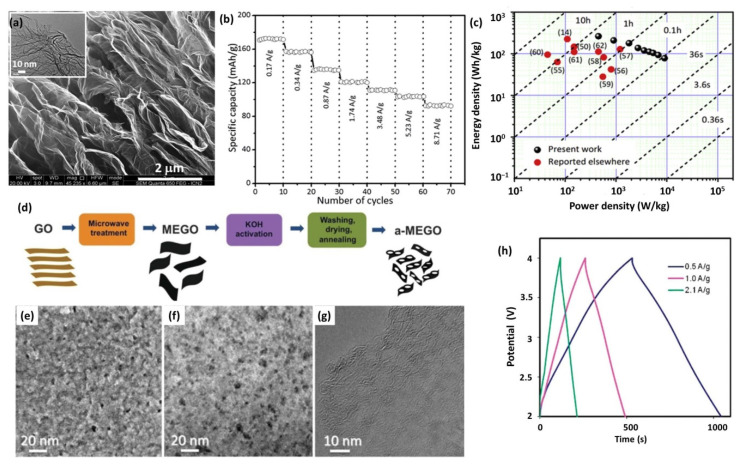
(**a**) FESEM of PRGO (inset shows TEM image); (**b**) Rate capability of PRGO; (**c**) Ragone plot for PRGO-based LICs. Reproduced with permission from Ref. [67]. Copyright 2018, Elsevier. (**d**) Schematic showing the microwave exfoliation/reduction of GO and the following chemical activation of MEGO with KOH; Images of (**e**) high-resolution SEM, (**f**) ADF-STEM and (**g**) high-resolution phase contrast electron micrograph of a-MEGO. Reproduced with permission from Ref. [110]. Copyright 2011, American Association for the Advancement of Science. (**h**) Charge/discharge curves for a-MEGO/graphite LICs at various current densities. Reproduced with permission from Ref. [123]. Copyright 2012, Royal Society of Chemistry.

Graphene oxide (GO) is an appealing precursor of graphene-based cathode. GO can be easily dispersed in polar solvent, due to the abundant oxygen-containing groups on the surface or at the edge [116]. Moreover, the oxygen-containing functional groups could provide extra capacity via redox reaction [111]. Generally, GO should be reduced by high temperature, hydrothermal reaction, or reductants to recover the conjugation structure before being used as cathode [66,67,117]. After the treatment, the reduced GO demonstrates enhanced electrical conductivity. Besides the typical double-layer adsorption/desorption charge storage, the remaining functional groups exhibit reversible Li^+^ binding and facilitate electrolyte infiltration, which largely improves the specific capacity and rate capability [66]. Several reports have explored the application of reduced GO-based cathodes in LICs [118,119,120,121]. As a typical example, Dubal et al. prepared a partially reduced GO (PRGO) by thermal annealing [67]. The obtained sample presented three-dimensional (3D) interconnected networks with open-porous nanosheets, which is expected to contribute a fast charge/discharge rate (Figure 4a). Moreover, the partial reduction strategy maintains a substantial amount of C=O redox groups, which could undergo redox reaction with consequent Li^+^ uptake [122]. Accordingly, PRGO shows an astonishing capacity of 171 mAh g^−1^, with an excellent rate of 92.3 mAh g^−1^ at 8.71 A g^−1^ (Figure 4b). The DC-LICs using PRGO as cathode deliver an ultrahigh energy density of 262 Wh kg^−1^, which keeps 78 Wh kg^−1^ at a high power density of 9 kW kg^−1^ (Figure 4c).

However, the reduced GO suffers from small SSA and poor conductivity because of the unavoidable sheet restacking and incomplete recovery of the conjugation structure, resulting in inferior electrochemical performance. Therefore, highly porous graphene cathodes with higher conductivity and larger SSA are developed by activation or the template-assisted chemical vapor deposition (CVD) method. As a pioneering work, Ruoff et al. developed a graphene cathode (a-MEGO) for LICs through the chemical activation of microwave-expanded graphite oxide to obtain a dense network of nanometer-scale pores surrounded by highly curved carbon layers (Figure 4d) [110]. As shown in Figure 4e–g, the a-MEGO demonstrated the porous morphology with a pore size distribution of sizes between ~1 and ~10 nm. More importantly, a-MEGO had a very high SSA of 3100 m^2^ g^−1^, while retaining the high conductivity of graphene. As expected, the a-MEGO cathode exhibited a nearly symmetric charge/discharge curve and delivered a specific capacitance of 266 and 213 F g^−1^ at 1.0 and 2.5 A g^−1^, respectively (Figure 4h). By pairing with graphite, the dual carbon-based devices presented a high energy density of 147.8 Wh kg^−1^ [123]. Similarly, Zhang et al. fabricated porous graphene by activation of reduced GO [124]. The obtained product had a highly crumpled morphology and porous structure with abundant mesopores that contributed to a high SSA (2103 m^2^ g^−1^) and large pore volume (1.8 cm^3^ g^−1^). This structure provides highly exposed active sites for ion adsorption/desorption and fast transport path for ions and electrons, leading to enhanced capacity and rate.

**Figure 5:**
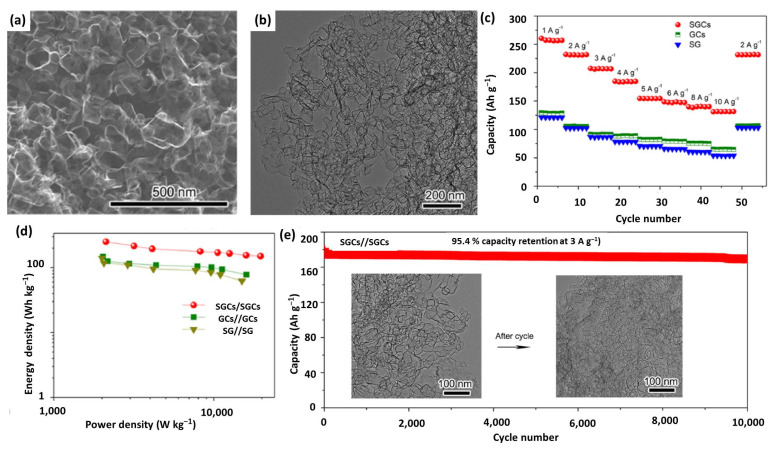
(**a**) SEM and (**b**) TEM images of SGCs; (**c**) Rate performance of SGCs cathode, compared with GCs and SG; (**d**) Ragone plots of SGCs//SGCs, compared with GCs//GCs and SG//SG; (**e**) Cyclic performance of SGCs//SGCs (the insets are the before and cycled TEM images of SGCs after 10,000 cycles). Reproduced with permission from Ref. [69]. Copyright 2022, Elsevier.

Besides chemical activation, porous graphene with high conductivity and outstanding structural stability could also be fabricated by chemical vapor deposition with the assistance of hard template [125,126,127]. For example, Xiao et al. synthesized S-doped graphene nano-capsules (SGCs) by CVD with the presence of a MgO template [69]. As displayed in Figure 5a,b, the SGCs exhibited an integrated nano-capsule structure with a uniform size of 50 nm, and no obvious cracked products were observed, indicating that SGCs have a good structural rigidity and stability. SGCs show an extremely high capacity of 257.1 mA h g^−1^ at 1 A g^−1^ and an appealing rate capability of 147.7 mA h g^−1^ at 6 A g^−1^, both of which are far better than those of the control samples (Figure 5c). With SGCs as both cathode and anode, the assembled symmetric LIC delivers an ultrahigh energy density of 249.9 Wh kg^−1^ at a high power density of 2.12 kW kg^−1^, which still retains 149.8 Wh kg^−1^, even at 14.99 kW kg^−1^ (Figure 5d). As can be seen from Figure 5e, the SGCs//SGCs also presents excellent long-term cycling stability with a retention of 95.4% after 10,000 cycles. It should be noted that the electrode still keeps an intactness and undamaged nano-capsules morphology, even after cycling for 10,000 times, verifying the superior structural stability of SGCs (inset of Figure 5e). The above excellent properties could be ascribed to the hollowed and stable structure with an abundant mesopore-dominant porosity, good electronical conductivity, enlarged interlayer spacing, and S-doping of SGCs.

**Figure 6:**
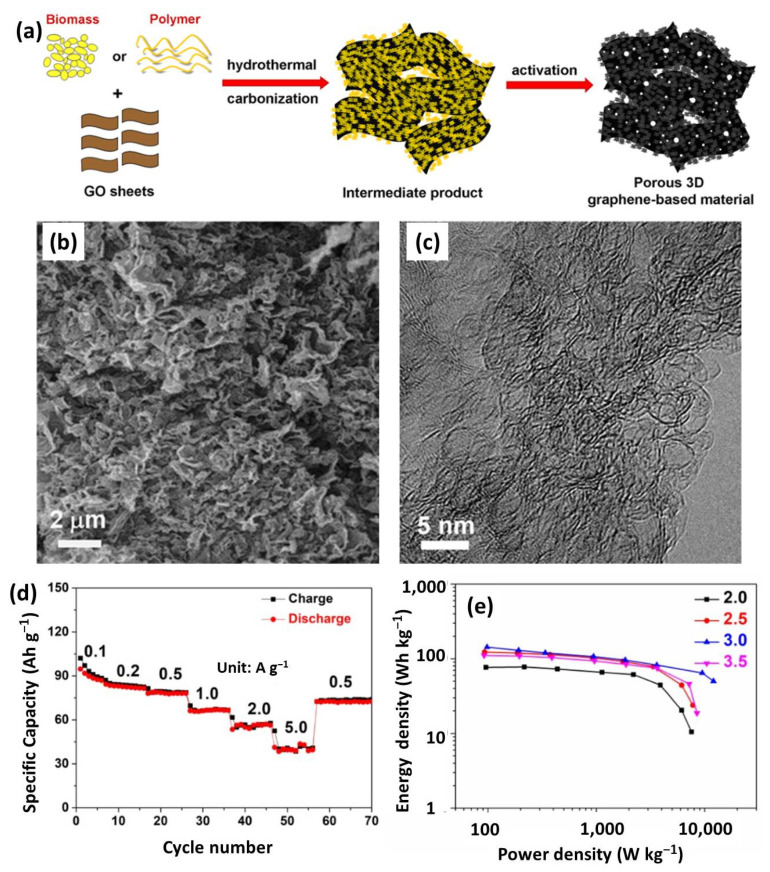
(**a**) A preparation schematic of porous 3D graphene-based materials; (**b**) SEM and (**c**) TEM images of porous 3D graphene-based products. Reproduced with permission from Ref. [13]. Copyright 2013, Springer Nature. (**d**) Rate performance of 3D graphene-based cathode; (**e**) Ragone plots of all-graphene-based LICs with different mass ratios. Reproduced with permission from Ref. [129]. Copyright 2021, IOP Publishing.

Despite the superb electrochemical performance for pure graphene-based cathodes, the high cost and tedious preparation procedure promotes researchers to explore graphene-based composites. This strategy overcomes the high cost of graphene, but keeps its inherently excellent properties. Moreover, forming composites with other materials could prevent the restacking and agglomerating of graphene. Chen’s group presented a simple and green, but very efficient, approach to prepare 3D graphene-based porous materials through in-situ hydrothermal polymerization/carbonization of the mixture of cheap biomass or industry carbon sources with GO, followed by chemical activation (Figure 6a) [13,128]. The optimal product presented a sponge-like morphology and porous microstructure (Figure 6b,c). More importantly, it had an ultrahigh SSA (3523 m^2^ g^−1^), mesopore-dominated porosity, and excellent bulk conductivity (up to 303 S m^−1^), thus contributing an outstanding electrode for SCs and LICs [24,33]. As shown in Figure 6d, the 3D porous graphene-based cathode demonstrated a high capacity and outstanding rate [129]. Benefiting from the dual graphene-based electrodes, the obtained all-graphene LIC with optimized cathode/anode ratio delivered a maximum energy density of 142.9 Wh kg^−1^ and a peak power energy of 12.1 kW kg^−1^ (Figure 6e). Anyway, heteroatom doping was applied to enhance the electrochemical performances of graphene-based materials. For instance, Wang et al. designed 3D porous activated nitrogen-doped graphene sheet (A-N-GS) by aniline polymerization with GO and then KOH activation [70]. A-N-GS demonstrated a sheet-like structure with a rough surface and 3D interconnected porous network. Coupled with the 3D highly conductive pathway and high-level nitrogen doping, A-N-GS showed a much improved capacity and rate performance, compared with the non-doped or non-activated samples. With a graphene-based anode, the all graphene LICs could reach an ultrahigh energy density of 187.9 Wh kg^−1^ at a power density of 2.25 kW kg^−1^, which still remained at 111.4 Wh kg^−1^, even at 11.25 kW kg^−1^.

#### 2.2.2. Carbon Nanotube-Based Cathode

As a typical one-dimensional nanosized carbon material, the carbon nanotube has exceptional conductivity, large aspect ratio, flexibility, and excellent mechanical strength [130]. All these merits make CNTs superior electrode material in various energy conversion and storage systems [131]. Particularly, CNTs are excellent substrates for fabricating flexible devices [132,133]. Nonetheless, similar graphene, CNTs usually demonstrate mediocre electrochemical performance, due to the severe agglomeration issue. As a consequence, forming composites with other materials is a feasible method, in which CNTs serve as either a spacer to increase the SSA or a conductive additive to enhance the electrical conductivity [134].

**Figure 7:**
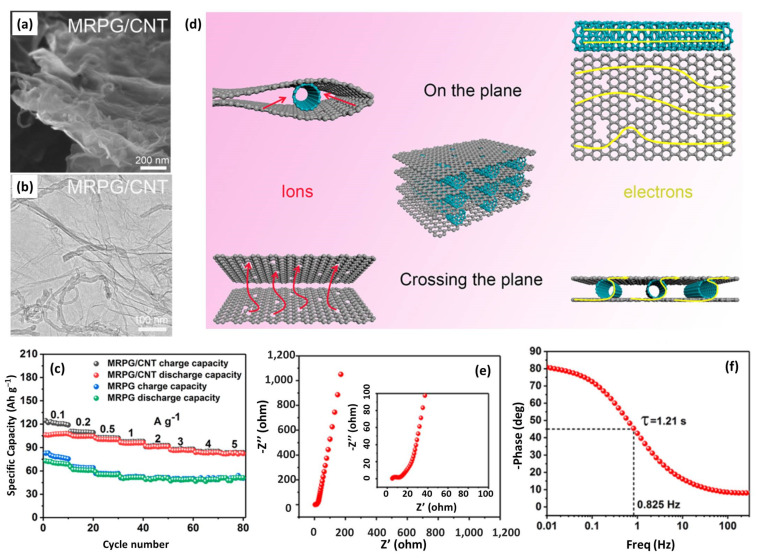
(**a**) SEM and (**b**) TEM images of MRPG/CNT; (**c**) Rate performanceof MRPG and MRPG/CNT; (**d**) Schematic illustration of the ion channels and electron channels in MRPG/CNT; (**e**) Nyquist impedance and (**f**) Bode plots of MRPG/CNT//MRPG/CNT LIC. Reproduced with permission from Ref. [71]. Copyright 2021, American Chemical Society.

Several reports have verified that CNTs/graphene composites can solve the restacking issue of both the two components and form a 3D conductive network [135,136,137]. Additionally, the increased SSA and the well-distributed nanopores promote electrolyte infiltration and diffusion, endowing the obtained product-enhanced electrolyte accessibility. Hence, the high capacity and excellent rate could be expected for CNTs-based composites. Bai et al. proposed a CNT supported porous graphene (MRPG/CNT) by a facile microwave irradiation method [71]. From the SEM and TEM images in Figure 7a,b, CNT was uniformly distributed between graphene sheets to support the layer structure. The obtained MRPG/CNT cathode showed much improved capacity and rate capability, compared with MRPG (Figure 7c). In addition, the DC-LIC with symmetric electrodes achieved a maximum energy density of 232.6 Wh kg^−1^ and an extremely high power density of 45.2 kW kg^−1^. The authors deemed that the excellent electrochemical properties could be explained by the 3D ion/electron channel model, as illustrated in Figure 7d. CNT intercalation into graphene sheets inhibited the restacking of graphene, expanded the layer space, and improved the electrode conductivity, forming a well in-plane and cross-plane channels for both the ion and electron migration. This model is supported by the Nyquist plot and Bode plot (Figure 7e,f). MRPG/CNT//MRPG/CNT LIC has a small equivalent series resistance of 18.0 Ω, which is beneficial for high rate of output and long-term cycling. Furthermore, the characteristic relaxation time constant τ was calculated to be 1.21 s, indicating the fast reaction kinetics and high-power capability of the full cell.

Overall, graphene/CNT-based cathodes have advantages in achieving high capacity and outstanding rate performance. However, nanosized carbons usually have relatively lower density (~0.3 g cm^−3^) than commercial AC (~0.5 g cm^−3^) because of their low tapping density, resulting in actually the same or even lower volumetric energy density than commercial ACs [138]. To overcome this dilemma, practical graphene technologies, such as the capillary drying process and rapid drying process, provide a promising solution to fabricate high tap density graphene-based composites [139,140,141].

## 3. Progresses of Carbon Anode Materials for DC-LICs

As the battery-type electrode for DC-LICs, the anode plays a crucially important role in increasing energy density. On the other hand, both the power output and cycling stability are highly influenced by the anode, which are still the bottlenecks for advanced DC-LICs. Hence, developing anode materials with high capacities, high rates, and stable structures is of great significance to realize practical DC-LICs. As summarized in Table 2, carbon materials ranging from traditional carbon (graphite, hard carbon, soft carbon, etc.) to nanosized carbons (graphene, carbon nanotubes, graphdiyne, and their composites) have been applied as anodes.

### 3.1. Traditional Carbon Anode

#### 3.1.1. Graphite

Graphite is the mainstream anode for the current LIBs industry, owing to its low cost, excellent electrical conductivity, and relatively high capacity. In addition, graphite with a long-range ordered structure has a nearly flat charge/discharge profile at low potential vs. Li/Li^+^, which thus enables DC-LICs with a high working voltage by combining the graphite with a capacitor-type cathode [62,75,77,154]. Considering the relatively high taping density, graphite has the advantage of achieving high volumetric capacity. Recently, recycling graphite from spent LIBs has become an important research topic, owing to sustainability and low cost [155,156,157]. For instance, Aravindan et al. proposed an efficient route to reutilize the recovered graphite (RG) as the anode to fabricate DC-LIC [158]. After a simple treatment, the RG demonstrates a comparable capacity and rate with pristine graphite. The obtained full cell delivers a maximum energy density of 185.5 Wh kg^–1^, with excellent low/high-temperature performance.

Although graphite demonstrates appealing properties as an anode for DC-LICs, pristine graphite is not suitable for high-rate and long-cycling DC-LICs, due to the sluggish kinetics of the intercalation reaction and ion diffusion [159]. To achieve high-rate graphite, reducing the particle size and defect engineering are two typical strategies. For example, Pandolfo et al. evaluated the rate capability of various commercial graphite materials and confirmed that reducing the graphite particle size showed enhanced rate capability, but increased the irreversible capacity loss. [160]. Microcrystalline graphite (MG) consists of many closely packed graphitic nanocrystals with small inter-particle voids [161]. This structure enables MG with fast ion diffusion of nanostructured materials and high mass density of bulk materials simultaneously. MG is regarded to break the limitations of the slow Faradaic reaction and Li^+^ diffusion of bulk material, which well-matches the cathode by providing an ultrafast capacitor-like response. Hence, Kang et al. adopted high-temperature-purified MG as anode in LICs [162]. As expected, the MG-based LICs showed an extremely high power density of 352 kW kg^–1^. Chen’s work demonstrated that intrinsic lattice defect engineering could effectively enhance the fast-charging capability of the graphite [163]. The graphite thermally treated in CO_2_ had a superior rate capability of 209 mAh g^−1^ at 10 C (only 15 mAh g^−1^ for the pristine graphite), which is attributed to the defect-induced enhancement of the kinetics and pseudocapacitive storage.

#### 3.1.2. Hard/Soft Carbon

Besides graphite, hard carbon (HC) and soft carbon (SC) are two important anodes in high-power LIBs, which also show great potential in DC-LICs [60,164,165]. HC and SC are commonly prepared by the thermal pyrolysis of carbon-rich precursors [166]. Hard carbon, known as nongraphitizable carbon material, consists of a large fraction of curved and randomly distributed graphitic sheets and cannot be reshaped into graphite, even at a temperature of 3000 °C, because of the interlayer cross-linking of the precursors [167,168]. Notably, the disordered structure of HC allows for Li^+^ insertion on both sides of the graphene sheets within the microscopic graphite-like regions, leading to a potentially higher capacity [97]. Soft carbons, on the other hand, contain a semi-graphitic structure and have relatively fewer defects and higher crystallinity than HC [169]. Particularly, the typical structure of SC consists of a high strain region and a low strain region. The former is a disordered region with unorganized carbon, while the latter is a graphitic region with high crystallinity carbon. This novel structure enables SC with an excellent ion diffusion rate and high conductivity. Normally, HC and SC exhibit better rate performance and long-term cycling stability, due to their enlarged interlayer distance and increased electrode/electrolyte interface. Therefore, they have been recognized as promising anodes for high-power and long-life Li/Na/K-ion batteries and LICs [48,49,167,168,170].

Particle size has a profound effect on the rate capability of HC. For example, HC was prepared through simple polymerization of furfuryl alcohol at room temperature, followed by a calcination step [97]. To realize the desired high power, ball milling was performed on the HC to achieve a sub-micron particle size. In addition, the morphology of HCs can largely affect the electrochemical performances of LICs. By comparing the physical and electrochemical behaviors of spherical HC and irregular HC, Shi et al. found that irregular HC presented a distinct Li^+^ intercalation plateau at a low potential [76]. This helps the sufficient utilization of AC cathode and the cycling stability of LICs. Balducci et al. firstly proposed to apply petroleum coke-based SC in LICs [171]. The prepared sample had a moderate capacity of 250 mAh g^−1^ at 0.1 C, but a better rate capability than that of many graphite electrodes at high current densities. Later, the same group examined the cycling stability of petroleum coke-based LICs [172]. When operating at a working voltage of 4.0 V, the device could maintain an extremely stable cycling life of 50,000, with energy and power of 48 Wh kg^–1^ and 9 kW kg^–1^, respectively.

**Figure 8:**
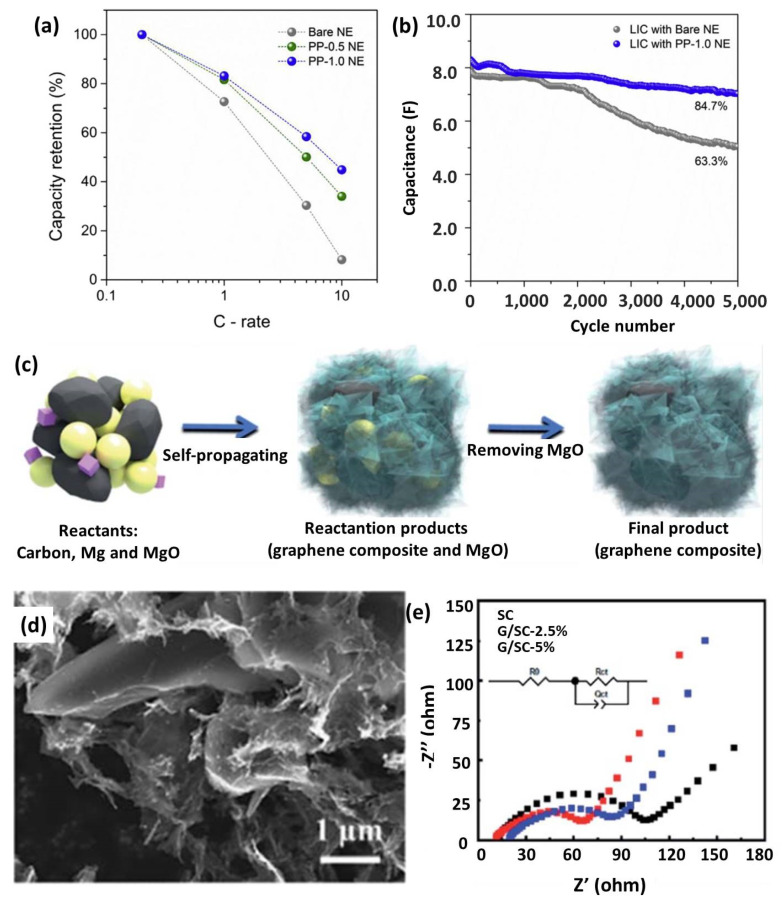
(**a**) Capacity retentions at various current densities during charge; (**b**) Capacitance retention of LICs employing bare NE and PP-1.0 NE. Reproduced with permission from Ref. [173]. Copyright 2015, Elsevier. (**c**) Schematic diagram of preparing G/SC; (**d**) SEM image of the G/SC composite; (**e**) EIS curves of pure SC and G/SC. Reproduced with permission from Ref. [78]. Copyright 2021, Royal Society of Chemistry.

Compared with the graphite, however, HC and SC have relatively lower electrical conductivity, owing to their disordered structure and abundant defects, which is detrimental to rate performance. Forming composites with conductive polymers or low-dimensional nanocarbons is an effective strategy to overcome this drawback. For example, Park et al. demonstrated that the incorporation of electrically conductive poly(3,4-ethylenedioxythiophene)-poly-styrene sulfonate (PEDOT-PSS) into soft carbon had a positive effect [173]. Thanks to the lowered electron and charge transfer resistance, the SC anode exhibited an enhanced charge capacity retention of 64% at 5 C with only 1.0 wt% PEDOT-PSS added (Figure 8a). In addition, better conductive network leads to an enhanced utilization of the electrode material, which can be observed from the higher capacity retention after prolonged cycling (Figure 8b). Numerous reports have verified that graphene could significantly improve the electrochemical performance of the composites by increasing conductivity and reducing contact resistance [93,122]. Ajuria et al. prepared a composite of recycled olive pit-derived hard carbon embedded in a reduced GO matrix (HC-rGO) [92]. The graphene sheets well-wrapped the HC particles and formed a 3D interconnected carbon network. This structure facilitated the electrolyte diffusion and enhanced the conductivity. The electrochemical impedance spectroscopy (EIS) analysis revealed that charge-transfer resistance at the electrode–electrolyte interphase and the charge transport resistance within the electrode were considerably decreased when adding reduced GO. Consequently, HC-rGO anodes displayed a much-improved rate capability, compared with bare HC, and LICs-based on them had higher energy density at high power density. Ma et al. designed graphene/soft carbon (G/SC) composites through a large-scale SHS strategy, which combined the advantages of high conductivity of graphene and the low cost of SC (Figure 8c) [78]. As shown in Figure 8d, SC was evenly coated by graphene, leading to enhanced reaction interface and electrical conductivity. The G/SC showed a reversible capacity of 360 mAh g^−1^ at 0.1 A g^−1^, which remained at 200 mAh g^−1^ at 4 A g^−1^ (38% higher than that of pure SC). The EIS result also verified that the reaction resistance was decreased by the additional interface area provided by the SHS-prepared graphene network (Figure 8e). Based on the above discussions, introducing highly conductive materials is a facile method to further enhance the performance of traditional carbon anodes.

In summary, conventional carbon anodes have received intensive research interest in the early study of DC-LICs because of their low cost, mature manufacturing process, and successful application in commercial energy storage devices. However, all of them have limitations. The sluggish reaction kinetics of graphite, unsatisfactory capacity of SC, voltage hysteresis, and large irreversible capacity of HC are still the main obstacles before large-scale commercialization. Possible research directions might be particle size reduction, interlay distance enlargement, and/or compositing with nanostructured carbons.

### 3.2. Nanosized Carbon-Based Anode

Nanosized carbons, such as graphene, CNTs, and graphdiyne, have been investigated as anodes in LIBs, owing to their unique structural, mechanical, and electrical properties [174,175]. The long-range π–π conjugation structure of nanocarbons ensures highly intrinsic conductivity, while the adjustable layer distance, tailorable porosity, and rich surface chemistry provide easy electrolyte penetration and fast ion diffusion rates. These superior properties enable them to have a high capacity and excellent rate capability. Moreover, nanocarbons can serve as effective buffering components or backbones in anode materials, benefiting the structural integrity and long-term cycling stability. Considering the sluggish kinetics of graphite, high irreversible loss of HC, and low specific capacity of SC, novel nanocarbon-based anodes provide an appealing alternative for DC-LICs.

#### 3.2.1. Graphene-Based Anode

Benefiting from the 2D structure and adjustable interlay distance, both sides of graphene sheets can host lithium ions, while the abundant in-plane defects and exposed edges of graphene further offer more active sites [176,177]. Coupled with high conductivity, graphene has been regarded as an advanced anode with high specific capacity and fast charge/discharge rates for LICs. As a pioneering work, Zhou et al. adopted the pre-lithiated graphene nanosheets as anode materials and compared them with conventional graphite [77]. The graphene anode has a wide pore size distribution, with the co-existence of micropores and mesopores, which are responsible for charge storage and ion transport from electrolyte to electrode interface, respectively. Benefiting from the reduced charge transfer impedance and enhanced Li^+^ diffusion rate, the full cells based on graphene anode have higher energy and power densities than those of graphite-based devices.

**Figure 9:**
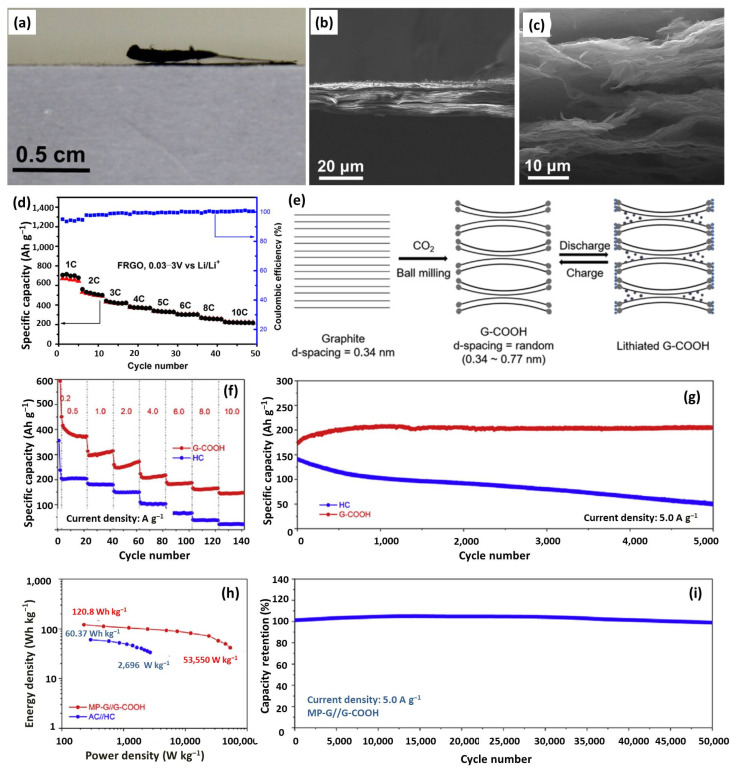
(**a**) Optical image of side view of partially reduced GO film disc; Side view SEM images of GO film (**b**) and FRGO film (**c**); (**d**) Rate performance of FRGO. Reproduced with permission from Ref. [18]. Copyright 2015, Elsevier. (**e**) Lithium-ion storage mechanism scheme of G-COOH; (**f**) Rate and (**g**) cycling performances of G-COOH and HC; (**h**) Ragone Plot and (**i**) long cycling performance of MP-G//G-COOH. Reproduced with permission from Ref. [127]. Copyright 2019, Elsevier.

Binder-free, self-supporting graphene films are regarded as promising anodes for DC-LICs, due to the adjustable interlayer distance and their potential to prepare flexible devices. By multiple exposure of a free-standing GO film in a focused camera photoflash, Huang et al. prepared a flash-reduced GO (FRGO) [18]. As shown in Figure 9a of the side-view optical image, the thickness of the flash-reduced part expanded distinctly, compared with the unreduced GO film. This could be further verified by the cross-sectional SEM images (Figure 9b,c). The GO sheets of the pristine film were tightly stacked, while the FRGO showed a quite swelled structure with large voids and pathways formed between the highly warped graphene. The increased pressure between the GO sheets during thermal heating-induced de-oxygenation accounts for the loose and open-pore structure, facilitating a rapid Li^+^ diffusion and, thus, enabling FRGO with enhanced ion intercalation kinetics at high charge/discharge rates. FRGO has a reversible capacity of more than 660 mAh g^−1^ at 1 C and excellent rate of 220 mAh g^−1^ at 10 C (Figure 9d). Zheng et al. synthesized an anode of edge-carbonylated graphene nanosheets (G-COOH) with enlarged interlayer distance via a ball milling method [127]. As shown in Figure 9e, the charge storage abides by the following mechanisms: (i) the rich porosity enabling rapid lithium-ion diffusion and, thus, fast reaction kinetics; (ii) the high degree edge-carboxylation structure storing lithium ions via reaction between C=O and Li^+^; (iii) the carboxyl modification enlarging the graphite layer space for fast lithium-ion insertion and desertion. All the features endow G-COOH with fast pseudocapacitive and lithium-inserted capacity, as well as long-term durability, which are far better than HC (Figure 9f,g). Integrated with a porous graphene cathode, the assembled all-graphene LIC delivers an ultrahigh power density of 53.55 kW kg^−1^ and an unprecedented cycling stability of 98.9% retention after 50,000 cycles (Figure 9h,i). Based on the above discussions, the merits of tunable interlayer space, rich surface/edge chemistry, and high electrical conductivity endow graphene-based anodes with a high capacity and excellent rate capability. On the other hand, the high cost and tedious preparation process promote researchers to develop graphene-based composites.

**Figure 10:**
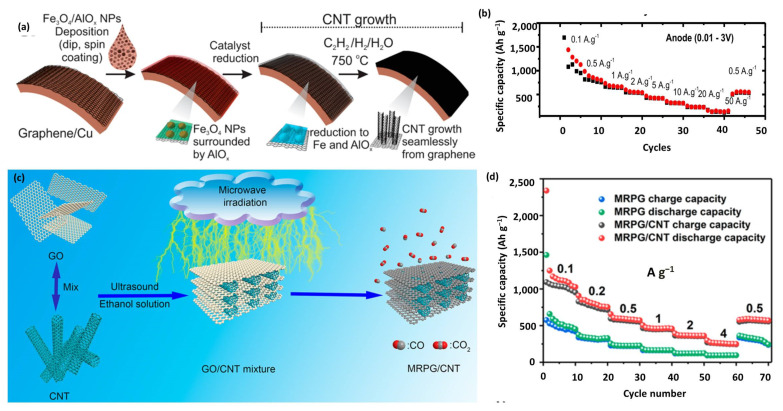
(**a**) Scheme for the growth of GCNT hybrid materials; (**b**) Rate testing of GCNT. Reproduced with permission from Ref. [137]. Copyright 2017, American Chemical Society. (**c**) Schematic illustration of the synthesis process of MRPG/CNT; (**d**) Rate performance of MRPG and MRPG/CNT. Reproduced with permission from Ref. [71]. Copyright 2019, American Chemical Society.

As discussed in Section 2.2.1, pure graphene has the disadvantage of unavoidable restacking and a high cost. Under these circumstances, CNTs are usually used as a spacer to prohibit the agglomeration of graphene [134,178]. Hydrothermal treatments or chemical reduction reactions are adopted to form graphene/CNTs composites [135,136,179]. However, the composites prepared by these two methods suffer from the insufficient reduction of GO and destruction of the CNT structure, leading to increased ohmic resistance. Consequently, Tour et al. seamlessly designed well-connected graphene CNT carpets (GCNT) through CNT growth from graphene substrate (Figure 10a) [137]. Thanks to the continuous electrical path from the active material to the current collector, the obtained binder-free GCNT anode has a high capacity and excellent rate capability (Figure 10b). By applying GCNT as both the anode and cathode, the obtained binder-free DC-LICs exhibited high energy density (∼120 Wh kg^−1^) and outstanding power density (∼20.5 kW kg^−1^). Another group reported a graphene-based electrode material via microwave irradiation of GO/CNT mixture (Figure 10c) [71]. The CNT intercalation into graphene prevented the restacking of graphene and enhanced the conductivity of the composite. Benefiting from a highly porous structure and better conductivity, MRPG/CNT showed a higher capacity and better rate performance than pure MRPG (Figure 10d). Specifically, MRPG/CNT showed high capacities of 1249 and 370 mAh g^−1^ at 0.1 and 2 A g^−1^, respectively.

#### 3.2.2. Graphdiyne-Based Anode

As a new 2D nanocarbon allotrope, consisting of a diethynyl group and a benzene ring, graphdiyne (GDY) was synthesized for the first time in 2010 by an in-situ chemical reaction of hexaethynylbenzene on a copper surface [180]. Stemming from its unique molecular structure with a high degree of π conjugation, GDY and its composites possess the characteristics of both a 2D material and a 3D porous material, such as high SSA, uniform porous channels, fast electron transport, wide electrochemical potential windows, and large double-layer capacitance [181,182]. GDY can store energy via two mechanisms, i.e., the Faradaic process in the bulk material and non-Faradaic process via reversible ion adsorption/desorption on the surface of pores [183]. All these features make GDY-based materials exhibit promising electrochemical properties in LICs.

**Figure 11:**
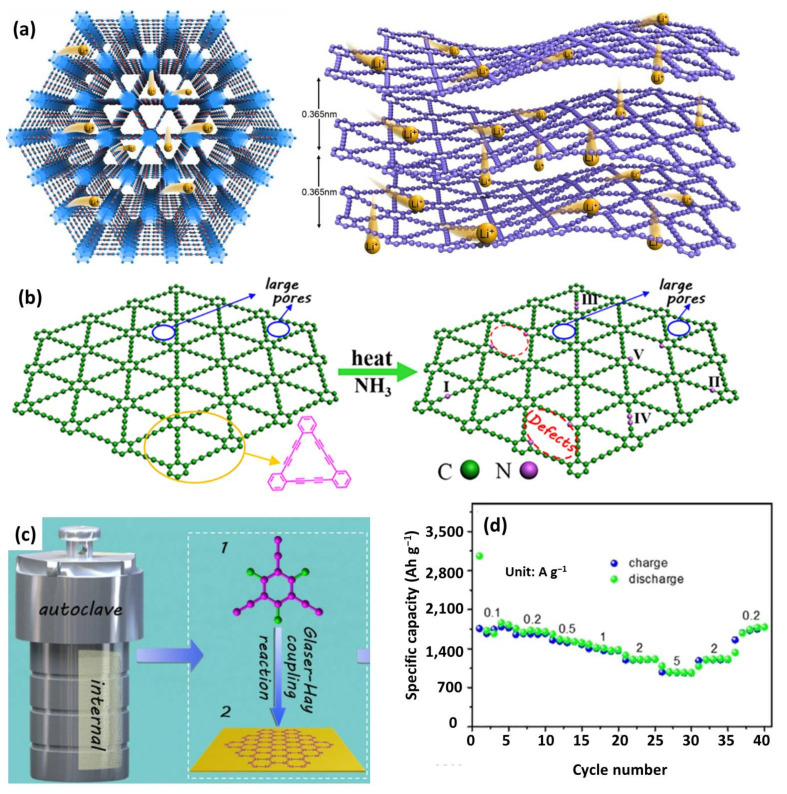
(**a**) Scheme of lithium diffusion in GDY layers. Reproduced with permission from Ref. [183]. Copyright 2016, Elsevier. (**b**) Schematic illustration of preparation of N-GDY. Reproduced with permission from Ref. [149]. Copyright 2019, Wiley-VCH. (**c**) The schematic of synthesis of F-GDY; (**d**) Rate capability of F-GDY. Reproduced with permission from Ref. [150]. Copyright 2018, Wiley-VCH.

Huang’s group firstly proposed using GDY as an anode in LICs by pairing with AC [183]. The 2D atomic layer structure coupled with abundant meso-/micro-pores enables GDY Li^+^ diffusion (both in-plane and out-of-plane), high electrolyte ion transport rate, and an abundance of electroactive sites (Figure 11a). As an anode, GDY delivers a moderate specific capacity of 572.5 (mAh g^−1^), and the LICs have acceptable energy density (112.2 Wh kg^−1^). To further improve the capacity, the authors prepared N-doped graphdiyne (N-GDY) by nitriding under NH_3_ (Figure 11b) and fluorine-enriched graphdiyne (F-GDY) by solvothermal reaction (Figure 11c) [149,150]. For example, F-GDY possessed an ultrahigh capacity of 1825.9 mAh g^−1^ at 0.1 A g^−1^ and outstanding rate capability of 979.2 mAh g^−1^ at 2 A g^−1^ (Figure 11d). F-GDY-based LIC released a maximum energy and power density of 200.2 Wh kg^−1^ and 13.12 kW kg^−1^, respectively. The astonishing electrochemical performances could be ascribed to the 3D porous structure, high conductivity, enlarged interlayer spacing, and enhanced wettability due to the incorporation of heteroatom dopants.

In general, nanosized carbons with novel structures have the merits of high capacity and well-matched rates with cathodes, making them very promising anodes for high-energy and high-power DC-LICs. Nevertheless, several problems should be carefully investigated and solved before their commercial utilization, including the large irreversible capacity and low Coulomb efficiency during the initial charge/discharge, low volumetric capacity/energy density, sophisticated synthesis process, and high cost. These nettlesome issues are even worse for graphene-based anode. Currently, compositing nanosized carbon with conventional carbons is emerging as a promising solution by combining their advantages and overcoming the shortcomings of both. Anyway, elaborately regulating the microstructure (porosity and SSA) is another strategy for balancing the electrochemical properties, aiming to suppress the side reactions, but keep their merits.

## 4. Summary and Outlook

As discussed in the previous sections, carbon materials demonstrate superior properties as electrodes in DC-LICs, due to their inimitable advantages of high electrical conductivity, tunable microstructure, and physical/chemical/electrochemical stability. Overall, traditional carbons have advantages in material preparation, cost, and abundant resources, while nanosized carbons, such as graphene-based materials, show superiority in capacity and rate. Benefiting from the novel cell configuration and hybrid energy storage mechanism, DC-LICs demonstrate superior electrochemical performances over LIBs and SCs and could bridge the performance gap between them. Hence, DC-LICs show great potential in the application scenarios where high energy density and high power output are both required.

Although DC-LICs have achieved great progress in material design and preparation strategies over the past two decades, it is still a difficult and sophisticated project to achieve high energy/power densities and long lifespans simultaneously. Especially, the mismatches in capacity, kinetics, and cycling life between the cathode and anode still remain. Anyway, there is a long way to go for scalable and efficient pre-lithiation technology. Additionally, studies in energy/thermal management systems are rare, and they are indispensable components for practical applications. Last, but not least, the practical application of DC-LICs needs to be investigated in depth, and more application scenario should be explored. Therefore, the following challenges should be thoroughly investigated and solved prior to the commercialization of DC-LICs.

(1) Developing a high-capacity carbon cathode. LICs are supposed to be able to deliver high energy and power densities. However, high energy density is always achieved at the cost of power density and vice versa, which is owed to the mismatched capacity and kinetics between cathodes and anodes. Hence, developing a high-capacity cathode is the top priority. To optimize the capacity of the carbon cathode, porous carbons with the desired microstructure, such as hierarchical porous carbon with large SSA and rational pore size distribution, should be given more attention. Furthermore, designing novel carbon and carbon-based composites with controlled morphologies and structures for high-capacity cathodes is an urgent need. For example, forming composites with traditional carbons and nanosized carbons is an effective method for further enhancing the SSA and conductivity of porous carbons

(2) Designing high-rate and long-lifespan carbon anodes. Poor rates and limited cycling lives are the two main drawbacks for the carbon anode, which are ascribed to the well-known sluggish Faradaic reaction and structure degradation during the repeated charge/discharge processes, respectively. Numerous reports have verified that elaborately designed nanostructure help to enhance ion diffusion and alleviate volume changes, while heteroatom doping is beneficial for enlarging the interlayer distance and improving wettability, which enables carbon anodes with improved rates and prolonged cycling lives. However, a large irreversible capacity and low Coulombic efficiency, owing to the high SSA and rich defects for nanostructured materials, should not be ignored. Developing carbon-based anode materials with optimal surface chemical properties, inner microstructures, and structural stabilities should be received more attention.

(3) Exploring feasible and efficient pre-lithiation technologies. With the large amount electrolyte consumption forming SEI at the anode surface, pre-lithiation is of great significance for maintaining high performance by compensating for the loss of active charge carriers and extending the working voltage. However, most of the pre-lithiation technologies are unsafe, time-consuming, or not cost-efficient. Therefore, developing safe and commercial-scale pre-lithiation technology is of critical importance for practical applications.

(4) Investigating advanced electrolytes. The stable electrochemical window of an electrolyte determines the working voltage and, thus, influences the energy and power densities of the obtained device. Moreover, the electrolyte stability also affects cycling performance. Generally, the continuous reduction of electrolytes at the anode can be effectively prohibited by forming SEI. However, oxidation by losing electrons at high potential can hardly be avoided, and the generated gas and byproducts lead to increased resistance. With this, it is imperative to investigate the anti-oxidized electrolyte additives. Anyway, other types of electrolytes, such as “water-in salts” and gel electrolytes, should be developed and applied in LICs, due to their high safety.

(5) Developing suitable energy/thermal management systems for LICs. Energy storage devices need the aid of energy/thermal management systems to provide stable and high-quality power. The current energy/thermal management systems for LIBs and SCs are not suitable for LICs because of their different electrochemical properties. Unfortunately, very few reports about LICs have covered this topic. This is a very important research direction for practical applications to which both academia and industry should pay more attention.

(6) Other dual-carbon-based metal-ion capacitors. Lithium faces big obstacles, due to its limited reserves and uneven distribution, promoting researchers to investigate other systems. Sodium/potassium-based hybrid capacitors are deemed to be the most competitive candidates because they share similar electrochemical performances and working mechanism with LICs, but have a huge advantage in natural abundance. Anyway, novel multivalent metal-ion capacitors (such as Zn, Mg, Ca, and Al) have become the research hotspots, in that they have the potential to provide twice or triple the amount of electrons per unit of active materials and are less sensitive to air and water, not to mention their abundance. These systems offer a promising alternative to LICs and, hence, more efforts should be devoted to investigating electrode materials, electrolytes, device configurations, and energy storage mechanisms.

In summary, this review systematically overviews the recent developments of carbon cathodes and anodes, in which their physical and chemical properties, electrochemical performances, and advantages and disadvantages are discussed. DC-LICs have achieved remarkable progress, thanks to the extensive investigation of carbon electrode materials. Although many challenges still remain, we believe the drawbacks of current DC-LICs will be overcome with the cooperation of academic and industrial communities, and we hope this article could provide some guidelines for future researchers.

## Figures and Tables

**Table 1:** Typical carbon cathodes and the electrochemical performances of DC-LICs.

Carbon Cathodes			Performances of DC-LICs				Ref.
Materials	Specific Capacity (mAh g^−1^)	Rate Capability (mAh g^−1^)	Voltage (V)	Maximum Energy Density (Wh kg^−1^)	Maximum Power Density (kW kg^−1^)	Cycling Stability	
Commercial AC (YP-50F)	48	35 @ 1 A g^−1^	2.0−4.0	73.0	/	85% after 1000 cycles	[50]
KHPC−K	100.5	75 @ 10 A g^−1^	0.01–4.0	169.0	97.0	77.7% after 5000 cycles	[51]
NHCN-2	125	98 @ 15 A g^−1^	2.0–4.5	146.0	52.0	91% after 40,000 cycles	[52]
LPCs-3	80	26 @ 10 A g^−1^	2.0–4.0	97.0	11.4	92.3% after 5000 cycles	[53]
DPC-MK	149	69 @ 50 A g^−1^	1.0–4.2	160.6	39.7	95.6% after 8000 cycles	[54]
0.1-BNC	113	63 @ 10 A g^−1^	0.02–4.5	220.0	22.5	81% after 5000 cycles	[55]
S-NPC-40	95.9	43.2 @ 10 A g^−1^	0–4.0	176.1	20.0	82% after 20,000 cycles	[56]
CHPC	132	100 @ 8 A g^−1^	0.01–4.2	220	66.9	70% after 3000 cycles	[57]
N/S-CNF0.25	133	102 @ 10 A g^−1^	1.0–4.3	154.0	18.6	92% after 6000 cycles	[58]
PHNCNB	72	51 @ 10 A g^−1^	1.0–4.0	148.5	25.0	90% after 8000 cycles	[59]
NHPCS	74	64 @ 5 A g^−1^	0–4.2	151.0	10.7	96.3% after 3000 cycles	[60]
NCNs-2	115	62 @ 10 A g^−1^	0–4.5	218.4	22.5	84.5% after 10,000 cycles	[61]
BNC	75.2	51.4 @ 5 A g^−1^	1.0–4.0	115.5	10.0	71.6% after 2000 cycles	[62]
ANCS	113	67 @ 10 A g^−1^	0–4.5	206.7	22.5	86.6% after 10,000 cycles	[63]
NPCS-1	97.4	51.3 @ 10 A g^−1^	0–4.0	135.6	10.0	82% after 10,000 cycles	[64]
NPCNF	122	53 @ 100 A g^−1^	1.0–4.3	143.0	45.0	83.1% after 10,000 cycles	[65]
URGO	35	29 @ 1.1 A g^−1^	2.0–4.0	106.0	4.2	~100% after 1000 cycles	[66]
PRGO	171	92.3 @ 8.71 A g^−1^	0.01–4.0	262.0	9.0	91% after 4000 cycles	[67]
NGF-0	82	61 @ 8 A g^−1^	1.0–4.0	147	48.9	87% after 10,000 cycles	[68]
GPC	95	40 @ 5 A g^−1^	0–4.2	142.9	12.1	88% after 5000 cycles	[18]
SGCs	257.1	147.7 @ 6 A g^−1^	0–4.0	249.9	19.62	95.4% after 10,000 cycles	[69]
A-N-GS	104	57 @ 5 A g^−1^	0–4.5	187.9	11.25	93.5% after 3000 cycles	[70]
MRPG/CNT	108	83.3 @ 5 A g^− 1^	0–4.5	232.6	45.2	86% after 5000 cycles	[71]
Zn_90_Co_10_-APC	118.8	50 @ 5 A g^− 1^	2.0–4.0	108	15.0	86% after 10,000 cycles	[72]

**Table 2:** Typical carbon anodes and the electrochemical performances of DC-LICs.

Carbon Anodes			Performances of DC-LICs				Ref.
Materials	Specific Capacity (mAh g^−1^)	Rate Capability (mAh g^−1^)	Voltage (V)	Maximum Energy Density (Wh kg^−1^)	Maximum Power Density (kW kg^−1^)	Cycling Stability	
HC	423	100 @ 50 C	1.5−4.2	110.0	25.0	81% after 10,000 cycles	[97]
HC-rGO	450	162 @ 10 C	1.5−4.2	130.0	5.5	88% after 10,000 cycles	[92]
PHC-4	1040.2	231.7 @ 6.4 A g^−1^	2.0−4.0	104.0	11.9	84.7% after 5000 cycles	[142]
KHPC-600	1064	280 @ 10 A g^−1^	0.01−4.0	169.0	97.0	77.7% after 5000 cycles	[51]
HNBC	1392	620 @ 1 A g^−1^	0−4.5	186.31	11.25	81.9% after 10,000 cycles	[143]
G/SC	360	200 @ 4 A g^−1^	2.0−4.0	151.0	18.9	93.8% after 10,000 cycles	[78]
SLC	829	148 @ 10 A g^−1^	0−4.0	127.0	33.57	99% after 100,000 cycles	[91]
NOPCNS	810	249 @ 50 A g^−1^	2.0−4.2	184.0	78.1	70% after 10,000 cycles	[144]
GOCAF	398	195 @ 10 C	1.5−4.2	100.0	9.0	80% after 15,000 cycles	[93]
NOPCNS	810	249 @ 50 A g^−1^	0−4.0	184.0	78.1	70% after 10,000 cycles	[144]
HNBC	1392	300 @ 5 A g^−1^	0−4.5	186.31	11.25	81.9% after 10,000 cycles	[143]
NPC	1740	369 @ 10 A g^−1^	0−4.5	203	90.0	80% after 20,000 cycles	[145]
HNCNBs	850	321 @ 20 A g^−1^	1.0−4.0	148.5	25.0	90% after 8000 cycles	[59]
BDC	1018	564 @ 5 A g^−1^	2.0−4.5	207.0	17.06	88% after 15,000 cycles	[146]
NDPC-0.5	1000	295 @ 5 A g^−1^	0−4.0	116.9	10.0	81% after 8000 cycles	[147]
FRGO	660	220 @ 3.72 A g^−1^	0−4.2	148.3	7.8	80% after 3000 cycles	[18]
PRGO	982	166 @ 20 A g^−1^	0.01−4.0	262.0	9.0	91% after 4000 cycles	[67]
PDA-GN	1150	371 @ 5 A g^−1^	0−4.2	135.6	21.0	65% after 3000 cycles	[124]
SHSG	854	333 @ 10 C	2.0−4.5	146.0	52.0	~91% after 40,000 cycles	[52]
NPG	859	758 @ 2 A g^−1^	1.0−4.0	195.0	14.98	~100% after 5000 cycles	[148]
G-COOH	450	145 @ 10 A g^−1^	1.0–4.2	120.8	53.55	98.9% after 50,000 cycles	[127]
rGO800-P	461	185 @ 10 C	1.5−4.5	91.0	26.0	76% after 10,000 cycles	[117]
F-GDY	1825.9	979.2 @ 5 A g^−1^	2.0−4.0	200.2	13.117	80% after 6000 cycles	[149]
N-GDY	1096.1	440 @ 4 A g^−1^	2.0−4.0	174.0	11.25	89.7% after 2000 cycles	[150]
GC1100	354	222 @ 2 A g^−1^	2.0−4.0	104.0	6.628	96.5 % after 3000 cycles	[151]
GNS-13	356	66.7 @ 5 A g^−1^	2.0−4.0	112.0	19.6	96.5% after 5000 cycles	[152]
GMC	119	378 @ 1 A g^−1^	0−4.5	190.63	11.25	81.8% after 10,000 cycles	[153]
